# ‘Living at work’: COVID-19, remote-working and the spatio-relational reorganisation of professional services in UK universities

**DOI:** 10.1007/s10734-022-00892-y

**Published:** 2022-07-07

**Authors:** Richard Watermeyer, Cathryn Knight, Tom Crick, Mar Borras

**Affiliations:** 1 Present Address: School of Education, University of Bristol, Bristol, BS8 1JA UK; 2 School of Education, Swansea University, Swansea, UK; 3 Manchester Institute of Education, University of Manchester, Manchester, UK

**Keywords:** COVID-19, Remote working, Professional services staff, Spatio-relational impacts, Trust, Virtual connectedness, Future of work

## Abstract

The COVID-19 pandemic has been the source of large-scale disruption to the work practices of university staff, across the UK and globally. This article reports the experiences of *n* = 4731 professional services staff (PSS) working in UK universities and their experiences of pandemic-related work disruption. It specifically focuses on a transition to remote-working as a consequence of social restrictions and campus closures, presenting both quantitative and qualitative findings that speak to the various spatio-relational impacts of PSS working at distance from university campuses. These survey findings contribute to a new narrative of work organisation in higher education which addresses the potential of remote-working as a means for boundary crossing, social connectedness and trust relationships in universities in the immediate context and strongly anticipated post-pandemic future.

## Introduction

The physical closure of university campuses around the world, in response to the SARS-CoV-2 (COVID-19) global pandemic, instigated major changes in working practices and conditions for university staff. While recent studies have considered the impact of a transition to remote-working on *academic* staff caused by the pandemic (McGaughey et al., 2021; Shankar et al., 2021; Watermeyer et al., 2021b, c), scant attention has been paid to the impact on *non-academic* and/or ‘para-academic’ (Macfarlane, 2010) staff working in the ‘third space’ (Whitchurch, 2013) of universities’ professional services arms. Such an oversight is not without precedent, with much research literature on work in higher education settings neglecting study of the professional services (Whitchurch, 2018). Yet, those referred to in this article as ‘professional services staff’ (PSS) are a major constituent of the UK higher education community (HESA, 2021) playing a significant role in the operation of universities as complex organisations (Shattock, 2010).

Universities in the UK — as in other neoliberalised settings — demonstrate isomorphic tendencies in their single-minded pursuit of productive outputs and positional gains (cf. Stensaker & Norgard, 2001; Parker, 2011) that converge into a ‘national brand’ (Lomer et al., 2018). The heterogeneity of their staff membership and the embeddedness of occupational stratification means, however, that the experience of recent and ongoing change affecting the higher education sector will be varied and diffuse. As tends to be the case in market-competitive settings, those disposed of a greater stock of capital and accordingly better insulated against the impacts of crisis — work intensification, labour exploitation and job insecurity — will have experienced a version of change less arduous than those with inferior capital.

In the context of higher education as a prestige economy (Blackmore & Kandiko, 2011), PSS are characterised as less visible and thus, less agentic institutional actors (Szekeres, 2006). Though their role expertise has become increasingly specialised in recent years as universities have adopted more explicitly corporate behaviours, their knowledge contribution, beyond a service function, tends to be overlooked by academics (Allen-Collinson, 2009; Ryttberg & Geschwind, 2017; Feather, 2015) and they remain culturally detached (McNay, 2005; Szekeres, 2004; Wohlmuther, 2008), despite growing instances of work integration (Locke et al., 2016; Whitchurch & Gordon, 2017). While this separation is prominent, it is injurious to universities’ attempts to operate as agile and flexible, and moreover, humane places of work.

Bridging these two cultures in universities may be theorised as a process of ‘boundary crossing’ (Akkerman & Bakker, 2011) and of PSS entering, acclimatising to, and inhabiting the world of academics, and vice versa. Self-evidently, some boundaries may be unyielding and not easily traversed, others may be more brittle, or porous and scalable. In highly stratified work environments such as the university, the former tends to hold. A capacity to boundary cross may then depend upon the availability of a ‘boundary object’ (Star & Griesemer, 1989), an event or phenomenon of shared concern, that bonds two communities through their common interest and works as a catalyst of ‘relational cohesion’ (cf. Lawler & Yoon, 1996). We propose the pandemic, or more specifically, the transition to remote-working caused by the pandemic, to be such a boundary object, an experience shared by all those working in universities, yet we would anticipate, differently.

Unlike their academic counterparts, PSS have historically been denied the ‘privilege’ of working ‘off-campus’ and from home. Instead, opportunities for remote-working, as we will show, have been piecemeal and/or hard-won. Moreover, while academics complain of the incursions of new-managerialism (Deem, 1998) and the corresponding diminution of their work-based autonomy, this is arguably slight in comparison to PSS as individuals working under the contiguous gaze and instruction of management. Such unequal treatment of academics and PSS is also suggestive of unequal gendered representation in the UK’s higher education space, where university staff demographics reveal gender bias and a majority of female workers (62%) occupying PSS roles set against a majority of male workers (53%) occupying academic roles (HESA, 2021).

Within the following discussion, we consider the spatio-relational impacts of transitioning to remote-working, during COVID-19, as experienced by PSS — as a heterogeneous staff group (Szekeres, 2011) — within their immediate and wider professional services teams, and in the context of their interactions with academic faculty. Moreover, we identify and discuss wider professional and personal impacts to PSS brought about by a transition to remote-working. We propose that an analysis of the relational impacts of emergency remote-working through the COVID-19 pandemic will help steer what is currently a highly uncertain course for PSS, a vast majority of whom continue, at time of writing, to work from home and may only partially return in the long-term to any kind of pre-COVID working format (Crick, 2021; Irons & Crick, 2022). Our findings thus provide a key contribution to existing, and what will likely be prolonged, discussions concerning the future post-pandemic organisation of work within universities — not only in the UK but internationally — and other similarly large and complex public organisations.

We present a series of perspectives that talk to the impact of emergency remote-working, which reflect a dearth of consensus apropos the future organisation of work in professional service divisions. First, however, we consider both the spatial reorganisation of PSS working lives brought about by the pandemic as framed by a wider history of remote-working — one albeit that neglects PSS — and the implications of remote-working for social connectedness as a major determinant of a successful work organisation.

## The rise of remote-working

While the pandemic has focused a spotlight on remote-working and its inequalities (cf. Reuschke & Felstead, 2020), such concerns are not *sui generis*. Instead, remote-working practices have proliferated and normalised over the course of recent years and are attributed amongst other things to the reorganisation of labour into a ‘knowledge economy’, technological connectivity enabling work to be carried out irrespective of spatial and temporal borders (CIPD, 2020; Messenger & Gschwind, 2016), and demographic changes such as workforce feminisation and the increased participation of women — especially women with child dependents, for whom flexible and remote-working practices are necessary (cf. Felstead & Henseke, 2017).

Social exchange theorists view remote-working as governed by a principle of reciprocity (cf. Cropanzano & Mitchell, 2005), where employee benefits of, for example, flexible working patterns are reciprocated to the employer by means of employees’ enhanced productive effort (cf. Kelliher & Anderson, 2010). Border theorists correspondingly argue that reciprocity leads to the imbrication of work and home (Clark, 2000), role conflict (Reuschke, 2019) and the collapsing of boundaries that protect employees from over-working and resultant injury to their health and wellbeing (Crosbie & Moore, 2004; see also Eurofound, 2020). Notwithstanding this reciprocal trade-off, a positive correlation is made between remote-working and job enthusiasm and satisfaction. Felstead & Henseke (2017), for example, report that remote workers are more favourably disposed to their employers and exhibit higher levels of organisational commitment than their conventionally sited counterparts, which may be linked to more trusting management-employee relationships (Reuschke & Felstead, 2021). Enhanced autonomy for employees is also associated with increased productivity (Bloom et al., 2015), although the overall contribution of remote-working to productivity gain is highly variable and differentiated by employment sector, employees’ level of education (Bartik et al., 2020) and the extent of their domestic commitments and personal privilege (Brynjolfsson et al., 2020). Yet while opinion of the contribution of remote-working varies, noises from both industry (PwC, 2021) and government (cf. UK Government, 2021) are that remote/flexible working practices will continue to feature prominently — if mainly in hybrid form — and remain highly relevant in a post-pandemic milieu (see also McKinsey & Company, 2020).

## Work as social connectedness

Since the onset of the pandemic and a transition to remote-working in universities, many members of the UK higher education community have spoken of their strong desire to return to campuses, to see their colleagues in physical form and to enjoy again the kinds of social interactions, virtual platforms fail to accommodate. Working remotely has been considered, by many, a long way second best to in-person interactions and its rapid transition is claimed to have contributed to employee disengagement (Adisa et al., 2021) and weakened social relatedness within organisations (Delfino & van der Kolk, 2021).

The bonds of social connectedness and emotional ties cultivated between co-workers — often through informal and spontaneous interactions — so crucial, as Durkheim (1917 [2008]) claims to achieving ‘collective effervescence’, and we might add critical solidarity, may be difficult to maintain in virtual encounters where emotional readings between interlocuters are obfuscated by a poverty of communication cues, lags and latency in connectivity, and even the disembodying effect of blank screens. Yet, emotions experienced through collective work-based interactions are argued to be indispensable to the formation and maintenance of group identity (Mackie et al., 2008) and function to ensure group stability (Keltner & Haidt, 1999). Thus, a feeling of belonging and community within work contexts may be sacrificed where the cultivation of positive work relationships — that underpin co-workers’ accountability to each other and their connectedness to their employer (Blatt & Camden, 2007) — is undermined by the emotional sterility of ‘teleworking’ (Ory & Mokhtarian, 2006). Moreover, we might posit the difficulty of facilitating assemblies of larger groups in virtual spaces and orchestrating meaningful conversations that exceed dyadic interactions and a preoccupation with the ‘profane’, in addition to the challenge of practicing social skills that are indispensable to the management and mentoring of staff. Given these multiple challenges, it is perhaps not at all surprising to find some industry bosses attributing a situation of ongoing remote-working to the depletion of employees’ social capital, especially amongst those whose intra-organisational ties are weak (New York Times, 2020).

Nonetheless, despite assertions that ‘human beings are fundamentally and inextricably social’ (Gabriel et al., 2016), and a perceived threat of physical distance denying their fullest social expression, suggestions of remote-working culminating in workers ‘bowling alone’ (Putnam, 2000) may be unduly pessimistic and amaurotic to the affordances of virtual connectedness to social capital. In fact, there is a growing corpus of evidence linking the application of information communication technologies to the accumulation of social capital (cf. Spottswood & Wohn, 2020) even in the milieu of employees whose onboarding has been exclusively online (Clark, 2021). We might speculate, therefore, that online platforms are not so much wastelands of emotional interaction amongst co-workers as they are triggers of an emotional disposition uncommon to office settings. Bourdieu (1990), in such case, is helpful in theorising the transition to remote-working as disruption in the *field* of interaction within universities that is transformative to the *habitus* — ‘... the evolving process through which individuals act, think, perceive and approach the world, and their role in it’ (Costa, 2013: 1) — of their staff. A transition to remote-working for PSS, in such terms, also resonates strongly with what Costa (2015) has previously documented as the changing experience of academics as digital scholars and a tension involving new and old dispositions within universities. Consequently, we consider whether the spatial adjustments of remote-working and a transition to what are presented as emotionally sterile virtual platforms have resulted in PSS becoming further estranged from each other and their places of work. Or conversely, and perhaps against the grain of popular expectation, has such transitioning elevated the value of co-operation (cf. Gibbs & Kharouf, 2020) and provided new spaces for work convergence, enabled workforce harmonisation and aided the social capital of lower status and marginalised institutional actors culminating in stronger trust relationships (cf. Tierney, 2006)?

In the following pages, we seek to test the efficacy of social connectedness when practiced at distance — or rather *virtual* connectedness — and what may be lost or gained by the spatial dissolution of those sitting centrally, yet also, in terms of power and agency, sitting on the periphery of their institutions. We also consider how virtual connectedness corresponds to the renewal or dissipation of trust in university governance, specifically involving university managers and university staff, and the potential, therefore, of remote-working as socially empowering and reconciliatory or further estranging.

Thus, our study seeks to address how the COVID-19 pandemic has and continues to shape the working lives of PSS in universities where traditional working practices have dissolved and been replaced with a new spatio-relational arrangement. While it focuses on data specific to the UK higher education context, the universal experience of emergency (and longer-term) remote working during the pandemic for university staff, means our discussion is salient to an international higher education community, facing as many other sectors, an at least partial remote working future.

Through an online survey, we have sought to establish answers to three guiding research questions:

- RQ1: *What was the overall experience of PSS working remotely during the COVID-19 pandemic?*
- RQ2: *What were the spatio-relational impacts of emergency remote working for PSS during the COVID-19 pandemic?*
- RQ3: *What is the (post-pandemic) future of work for university PSS?*

## Methods

Data was collected via an anonymous online survey which was distributed in April 2021 and kept open for 1 month. The target population for the survey was PSS working in UK universities. Demographic questions determined whether respondents met this criterion. Those who did not were removed from the sample post hoc. The survey was distributed via professional mailing lists, social media and other online platforms, and with the assistance of two higher education trade unions. This convenience sampling method was not designed to capture a representative sample; rather, data was sought to illuminate general patterns and trends characterising the experience of UK professional services staff during the pandemic.

The online survey was designed and distributed via *Qualtrics*. Survey questions took inspiration from previous attitudinal surveys of the impact of COVID-19 on higher education communities (Watermeyer et al., 2021b, c) and piloted on a population subsample (*n* = 53). Feedback was gathered and the questions were refined before the survey was formally launched.

The survey consisted of *n* = 36 items including demographic and occupational questions, closed-ended questions seeking participants’ experiences and opinions on the impact of COVID-19 on their role and open-ended questions: ‘What benefits/negatives to your work life have you experienced as a result of the COVID-19 pandemic?’ (RQ1) (3972 responses 83.9%, mean number of words 22, s.d. 27); ‘Overall, how would you describe changes to your working relationships as a result of the pandemic?’ (RQ2) (3537 responses 74.8%, mean number of words 27, s.d.26) and ‘What do you think will be the long-term impact(s) of the pandemic on university professional services (if any)?’ (RQ3) (3415 responses 72.2%, mean number of words 23, s.d. 23). The large number of responses and average number of words for each open-text question confirm the engagement of respondents with the qualitative dimension of our study and the richness of the dataset, the analysis of which forms the major contribution of this article.

Descriptive statistics were employed to define overall trends in the population and to frame our analysis of the qualitative data. Open-ended questions were thematically analysed (Braun & Clarke, 2006). Responses were read and coded by an initial researcher before being validated by the whole research team (RW, CK, TK) (IRR = 0.87). The average duration of survey completion, once excluding outliers, was 13 min 43 s.

### Sample

In total, *n* = 4731 professional services staff completed the survey. Table 1 provides demographic information which reveals that respondents came from a wide variety of branches of professional services, most coming from ‘learning and teaching/academic support’ (19.2%). Other more prominently featured PSS branches (+ 10% sample representation) include ‘student support’ (16.7%), ‘libraries’ (12.1%) and ‘research/innovations/enterprise’ (11.8%). 79.6% of the sample had been working in the HE sector for more than 6 years. Seventy-eight percent of respondents stated being in full-time employment, 89% on permanent contracts and 64% stated being employed in pre-1992 universities. The majority of our respondents (62.8%) stated having no caring responsibilities. The sample features a gender bias with 71.1% female participation. As we have already mentioned, HESA (2021) data shows that 62% of the non-academic HE workforce (*n* = 191,440) was female (*n* = 119,600) in 2020/2021 — when this survey was conducted. Therefore, while our sample shows an overrepresentation of female respondents, it mirrors a systemic gendered bias.

**Table 1:** Respondent demographics

Variable	Category	Percent
*Gender*	Male	27.6%
	Female	71.1%
	Prefer to self-describe	1.2%
*Age*	18–25	1.5%
	26–35	17%
	36–45	27.3%
	46–55	31.2%
	56–65	21.3%
	66–75	1%
	75 +	0%
	Prefer not to say	0.6%
*University*	Pre-1992	63.7%
	Post-1992	27.5%
	Unsure	8.8%
*Years working in sector*	0–5	20.4%
	6–10	40.2%
	11–15	18.4%
	16–20	16.2%
	21–25	12.0%
	26 +	13.2%
*Branch of professional services*	Accommodation services	2.0%
	Commercial services	0.9%
	Employability	2.0%
	Estates and facilities	5.4%
	Finance	3.5%
	Human resources	2.3%
	International and partnerships	2.5%
	IT/AV	8.4%
	Learning and teaching: academic support	19.2%
	Libraries	12.1%
	Marketing and communications	3.0%
	Planning and operations	4.3%
	Recruitment and admissions	5.4%
	Research/innovations/enterprise	11.8%
	Student support	16.7%
	Other	15.2%
*Employment status*	Part-time	21.7%
	Full-time	78.3%
*Employment terms*	Fixed term	9.0%
	Permanent	89.7%
	Zero-hours	0.4%
	Other	1.0%
*Caring responsibilities*	Yes	37.2%
	No	62.8%

## Results

We first present a summary overview of quantitative findings taken from the survey and guided by our core research questions. This overview is intended as a framing device for discussion of our qualitative data, which forms the bulk of the rest of the article. We offer no substantive analysis of quantitative data, for instance multivariate analysis, preferring to save this for future discussion.

### Closed responses

#### RQ1 — What was the overall experience of PSS working remotely during the COVID-19 pandemic?

Survey respondents were asked how the pandemic had impacted various aspects of their role. While 66% stated that their role had become ‘more demanding’, the majority (53%) said that their work hours had stayed the same. Fifty-two percent said that working from home had made no difference to their ability to do their job, while the majority (47%) said that it had made them more productive.

Respondents were also asked ‘how have changes in your work due to the COVID-19 pandemic impacted on your mental and physical health?’ Fifty-five percent reported that the changes had a negative impact on their mental health (21% positive impact, 25% no impact) while 50% reported a negative impact on their physical health (24% positive impact, 26% no impact).

Respondents were also asked to ‘rate the competency of your university’s senior leadership through the pandemic (e.g. Vice-Chancellor, Pro-Vice Chancellor level)’ and the majority (64.1%) rated them as either ‘extremely competent’ or ‘somewhat competent’.

#### RQ2 — What were the spatio-relational impacts of emergency remote working for PSS during the COVID-19 pandemic?

Respondents were asked how working from home had impacted on how they are line-managed. Sixty-two percent stated no impact, while 23% of respondents stated being less closely monitored by line managers. Respondents were additionally asked about the impact of the pandemic on their working relationships with academic staff and their own team. The majority (57%) stated that their working relationships with academic staff had stayed the same, while 18% stated that they had improved, and 26% stated that they had deteriorated. Similarly, the majority of respondents (47%) stated that their working relationships with members of their team had stayed the same, while 22% stated that they had improved and 31% stated that they had deteriorated.

#### RQ3 — What is the (post-pandemic) future of work for university PSS?

When asked ‘do you have any concerns for the future of your job?’, 36% stated ‘yes’, 44% ‘no’ and 20% were ‘unsure’. Those who identified as male or who preferred to self-describe were significantly more likely to say ‘yes’ whereas female respondents were more likely to say ‘no’.

Respondents indicated that in the future, they would prefer a blended approach to working from home (71%). Fifteen percent would prefer to be exclusively home-based, and 11% exclusively campus based.

### Open text responses

We now turn to thematic analysis of qualitative data generated through our open-text questions, and guided by our overarching research questions. We do this so as to identify the relational benefits and drawbacks of remote-working experienced by PSS during the pandemic, in the hope of plotting a pathway towards the organisational future of their work in universities. Qualitative excerpts are attributed with the branch(es) of professional service (e.g. Research/Innovation/Enterprise), gender (male/female) and institutional contexts (pre/post-1992 institution) of each speaker.

### The spatio-relational benefits of remote-working

We start with what our survey respondents identified as the various positive contributions of remote-working, including boundary crossing by PSS both within and across institutional settings. Respondents began by discussing how remote-working had helped to dismantle status hierarchies endemic to university working cultures, and provided technologically facilitated and time-efficient opportunities for working with colleagues unavailable in a pre-pandemic milieu, which were, by extension, observed as opportunities for critical reflection and professional growth. Remote-working was observed for providing greater visibility and status to PSS:

I have built more and better relationships by having online video calls with colleagues I would never usually see, I feel there has been a levelling out in terms of hierarchy and opportunities to participate in things (e.g. I could never before afford to take time out to attend academic events I support, but I can follow them online and continue to monitor work, or dip in and out - this gives me a much greater understanding that I can bring into my work and develop processes). (PSS1: Multibranched, Female, Post-1992)

Remote-working was also viewed as transformational to meeting-management for PSS, allowing them to become more available and better informed, yet concurrently more agile participants of (academic-led) meetings, prioritising their time at points of specific relevance and interest. Our respondents thus attributed remote-working to redefining the parameters of their interface with academics, providing them greater exposure and thus potentially increasing their institutional capital and capacity to steer academic decision-making:

Typically, academics would not always invite research office staff to meetings because 75% of discussions are not relevant. Now, they invite us and we can stay on the call whilst doing something else and listening in and just 'joining in the conversation' as necessary. Similarly, as academics are working more with research proposals etc. on MS teams, we are more included in conversations that otherwise would have happened in corridors. This helps to pick up issues earlier. (PSS2: Research/ Innovation/ Enterprise, Female, Post-1992)

While remote-working was viewed by respondents as improving their availability to academics, it was also seen as enabling increased participation by academics in fora led by PSS. Comment was here made of the greater spontaneity, fluidity and time efficiencies of remote-working via virtual meeting platforms and the ease with which meeting convenors might multiply-attendance:

I now host meetings with academics remotely and have found these to be more effective than face-to-face meetings. Usually, the meeting can happen with a shorter notice period without needing to book locations, etc. Multiple people can join the meetings and leave the meeting without causing distractions as would occur in a face-to-face meeting. (PSS3: Research/ Innovation/ Enterprise, Female, Post-1992)

A switch to virtual meeting platforms instigated by the pandemic was also credited by respondents as an important step for universities, in terms not only of aspiring to build more cohesive internal communities, but enhance connectivity between multiple institutions. In looking to the near future and multi-university networks (cf. Aoun, 2018) and open innovation networks (Huggins et al., 2020), respondents spoke of remote-working as a catalyst for cross-campus mobility:

I work across 8 HEIs spread across the Midlands. One of the things I had been trying to implement was more virtual meetings to save on the time and cost of travelling for in-person meetings. Due to the travel restrictions put in place these meeting had to become virtual saving both time and money. (PSS4: Research/ Innovation/ Enterprise, Female, Pre-1992)

Visibility was a recurrent theme for our respondents who spoke not only of remote-working and a switch to virtual meeting platforms in terms of efficiency and (academic) capital gains but in terms of its humanising effect. A window onto co-workers’ domestic worlds, facilitated by virtual meetings, was seen as an opportunity to suspend work personas, and for PSS to embrace the cognate risks and vulnerability of being seen outside of the office context and accordingly, therefore, to a different, arguably more authentic, presentation of self. The, albeit inadvertent shared presentation of co-workers’ non-working lives — typically hidden or else screened in office settings — may be understood as a moment of revelation and emancipation from the choreography of performative work cultures that affect only partial self-declarations. Paradoxically, therefore, co-workers are seen to become, through forced self-disclosure, more vivid and real and knowable to each other in the virtual space. The virtual space may be thus rationalised as a locus of collective vulnerability engendering greater trust and cohesion amongst co-workers. Bared of their professional camouflage and in the strangely augmented reality of the virtual space, where the intrusions of homelife are manifold and relentless — children shouting, doorbells ringing, dogs barking — co-workers manifest, through a miscellany of fallibility, not as PSS colleagues but human beings. Here, ostensibly, a new form of relationality and/or kinship emerges carried by emotions of empathy and humility, and by recognition of co-workers’ shared humanity in the face of universal struggle:

I think working from home during a pandemic we all got a feel for each other’s lives outside of work. Everyone has become more understanding I think and kids popping into a Zoom call is just the norm now. We are seeing people as moms and dads, sisters and brothers etc other than work colleagues. (PSS5: Research/ Innovation/ Enterprise, Female, Pre-1992)

The common experience of struggle operates as a unifying factor in these accounts, through which tribal divisions may be seen to recede, a compulsion for social differentiation wanes, and the boundaries erected to assert and guard identities collapse. Far less a cause of separation, remote-working, in our respondents’ accounts is valued for bringing co-workers closer together, stimulating collegiality and shared resilience:

The team feels closer as we have had far more regular all-team meetings than we would have had on campus. We have bonded in adversity. (PSS6: IT/AV, Female, Pre-1992)

The opening of home to the gaze of co-workers makes conspicuous the constraints of working lives — and not just as relates to a home-work transaction — which most of the time in office settings may be hidden or intentionally non-disclosed. Instead, co-workers’ struggles are seen to affect compassion and kindness. A spirit of greater tolerance and even absolution is reported that defies the behavioural traits common to the university as a performance oriented and hyper competitive institution that champions individualism (PSS7). For some, the technology underpinning their interactions is viewed as a tool of conviviality (PSS8):

I think everyone now has a bit more empathy and patience in dealing with colleagues. We all know this has been a difficult time, and we are all aware that everyone is dealing with different challenges, which I think has made everyone a bit more forgiving and flexible. (PSS7: Research/ Innovation/ Enterprise, Female, Pre-1992)

I feel like my relationships with all my colleagues have improved as a result of the pandemic as people are so much more grateful for help and expertise, and the use of tools like MS Teams has made these interactions much less formal than they would normally be. (PSS8: Learning & Teaching/Academic Support, Female, Pre-1992)

### The spatio-relational drawbacks of remote-working

The reconciliatory and remedial qualities of transitioning to remote-working were not recognised by all our respondents. Some, in contrast, described a situation of dwindling contact, even communication blackouts, explained by interactions with colleagues becoming more formalised and less ‘fun’:

My team no longer speak to each other on a daily basis and even if I do get in touch with them, I get short answers and it’s not the same as we don’t seem to have the fun conversations we used to, and things go unanswered entirely. (PSS9: Learning & Teaching/Academic Support, Female, Pre-1992)

No longer being able to easily chat informally, i.e. ask quick questions or make comments to colleagues nearby. This type of communication can lead to other discussions and information being exchanged. In other words, communication between colleagues within our office has become, as a % of total communication, more formal. (PSS10: Research/ Innovation/ Enterprise, Male, Post-1992)

Respondents also spoke of their hesitance in reaching out to colleagues, where reaching out might be construed as an intrusion of time, reflecting thus, the intensity of remote-working and the absence of natural punctuations, the ebb and flow, and diversions inherent to office life. They viewed the unboundedness of remote-working as necessitating more socially conscientious and disciplined approaches to their encounters with colleagues. The decay of the home as a protected or ‘off-limits’ space, previously (at least in part) insulated from the incursions of work, was seen by respondents to necessitate greater sensitivity and selectiveness in making demands of colleagues’ time. Respondents spoke for instance of not wanting to burden colleagues with additional online meetings necessitated by the lack of informal interactions:

I’m reluctant to contact colleagues as it feels too intrusive to keep calling them because they are working at home. (PSS11: Female, IT/AV, Pre-1992)

In such accounts, working from home is represented as inhibitive to spontaneous and unscripted work interactions, previously tolerated in the office as a communitarian space and designation of time collectively owned. Instead, the home as a sovereign space and designation of time individually claimed and defended results in co-workers becoming more discriminatory and/or restrained in pursuance of each other’s time. Consequently, the home is conceived as a site of reduced access to and amongst co-workers, where despite associations of informality and the potential of uninterrupted connectivity, ambivalence apropos rights of access and ill-defined etiquette, results in work-based interactions becoming more formal and structured. Respondents spoke of remote-working as domestic incarceration, and linked physical immobility with social impoverishment:

“Living at work” and . . . ‘being stuck’ in one place with no interpersonal human interaction is stressful and at times soul-destroying - we are hard wired for human connection and much of my work entails building working partnerships and alliances. (PSS12: Multibranched, Male, Pre-1992)

Respondents also spoke of their experiences of being unable to disconnect from work pressures (PSS13) and of the inundatory nature of virtual platforms inhibiting anticipatory and reflexive work practices (PSS14), even causing them to consider leaving their posts without onward employment (PSS16). Respondents like PSS15 also expressed their view of remote-working as an uneven ‘reciprocal’ arrangement (Cropanzano & Mitchell, 2005), which provoked feelings of anxiety and a tendency to over-compensate for the alleged benefits of working from home:

I have sometimes struggled to keep my “work-space” divided from my “home space” and never feel like I can fully leave work behind at the end of the day. (PSS13: Collaborative outreach, Female, Pre1992)

Use of Teams is out of control. I spend my day leaping between conversations. I’m always ‘on’ and available. No time to think about what I’m doing. Just react, react, react. (PSS14: IT/AV, Male, Post-1992)

The notion of 9-5 is obliterated as you feel you need to be on call or respond as soon as possible. Add in work-related guilt and anxiety because you are at home and comfortable, so you work harder, are always available as you have no other barriers to protect you. (PSS15: Educational Development, Female, Pre-1992)

I’m not sure I can continue to carry on at the pace and intensity, especially as it is likely to continue online for at least some of my role. I have (and am) contemplating leaving without anything to go onto. (PSS16: Employability, Male, Pre-1992)

While respondents, as previously discussed, spoke of the contribution of remote-working in challenging status hierarchies in universities, they also described them having become further entrenched due to general work intensification caused by the pandemic. Managing the demands of work intensification for PSS (and their status inferiority) was seen to be especially problematic in the context of reduced work resources and staff capacity as universities, in response to the economic challenges of the pandemic, implemented cuts to their operational budgets. Such cuts were attributed to the souring of relations between PSS and academics with the former inadequately equipped and becoming less resilient in handling the increased demands, (unreasonable) expectations and escalating ire of the latter:

Our capacity is being reduced as contractors and fixed-term staff aren’t being extended at the end of their contracts and the recruitment freeze means there is no one to replace them. Having to bear the brunt of academic staff’s frustrations is extremely challenging - we have often been asked to do the impossible but when we explain that the technology doesn’t exist we’re told that we’re incompetent or lazy and need to find a way to make it work. The way that some academic colleagues talk to us is totally unacceptable, but we’re just expected to take it. (PSS17: IT/AV, Female, Pre-1992)

Yes, academic staff are stressed and over-worked—but so is everyone else! I have definitely seen an increase in rudeness in the tone of emails and demands from academic staff to professional services staff. (PSS18: Research/Innovation/Enterprise, Male, Pre-1992).

In the context of remote-working, and therefore distanced power relations, there lies the danger that micro, or indeed cyber aggressions directed towards PSS become more commonplace and also, potentially, normalised where online moral disengagement proliferates (Runions & Back, 2015) without sanction. The work abuses and inequities potentially suffered by PSS as a subaltern constituency or as Watermeyer and Rowe (2021) have described a ‘massive minority’ in universities, are therefore prone to increase and particularly so where their work becomes rebundled into discrete online services which further subordinates their institutional status:

Mostly, I don’t feel academic staff are colleagues anymore: it feels like I am working in a shop and they are customers along with the students. I think that’s the way things were going anyway, with a move towards administrators working in separate places rather like an old-fashioned typing pool, and this has speeded things up. (PSS19: Learning & Teaching/Academic Support, Female, Pre-1992)

Also intimated in PSS19’s testimony is the debilitative impact of remote-working on weak ties and the relational compartmentalising prevalent to virtual connectivity that denies the potential for serendipitous encounters that catalyse weak ties and the overall development of social capital spanning an organisation’s network of employees (cf. Deal & Levenson, 2021).

### A hybridised future versus a return to the past?

Over 71% of our sample of *n* = 4731 respondents stated that in future they would prefer a blended approach to working; a percentage that rises to 76% amongst female respondents. Only 11% of the same number sought a return to a pre-COVID paradigm of full (or solely) campus-based working. In contemplating a model of hybrid working, respondents addressed the opportunity cost of working from home, in the context of campuses remaining the operational nerve-centre of universities and ‘in-person’ working remaining the preferred *modus operandi*, at least from a management perspective. They discussed the dangers of hybrid working in segmenting and therefore territorialising and tribalising (cf. Becher & Trowler, 2001) the PSS workforce into those who work from home and those who work on campus, and the potential therefore for a fractured, imbalanced and unequal community of workers:

I worry that there will be a division between those roles that can work from home (remotely) and will be able to do so in the future and those roles that will need to be on site to carry out their roles. This will need careful consideration by the leadership . . . If working remotely becomes the norm, how will they get involved/be included in university life at all? It is already so limited now. (PSS20: Administration, Female, Post-1992)

Conversely, respondents addressed concerns of institutions reverting to pre-COVID working practices, having seemingly embraced the affordances of remote-working while having identified drawbacks to working on campus. They spoke of the benefits of remote-working in corollary to the disadvantages of campus-based working. Moreover, the disadvantages they articulated that once may have been tolerated are now viewed as anathema. The experience of remote-working has in such terms made visible the costs of pre-COVID campus-based working lives and emboldened future imaginaries of work, even as might exist beyond the university. In fact, the experience of remote-working — and improved work autonomy — appears threatened in respondents’ accounts and their anticipation that freedom to work during the pandemic will be displaced and erode with the reassertion of managerialism over their working lives:

I am concerned about being forced back to working on campus in an unsuitable office and that this will mean I will lose any of the improvements over the last year. Its actively made me think of changing my job to something more local should this be the case. (PSS21: Learning & Teaching/Academic Support, Female, Pre-1992)

I’m concerned that the senior leadership team will expect all staff to go back to working on campus as we did pre-pandemic. The pandemic has shown me that this is not tenable for my mental and physical health in the future, and I will need to look very seriously at leaving my job to work somewhere closer to home if suitable blended working arrangements are not put in place. (PSS22: Research/Innovation/Enterprise, Female, Pre-1992)

## Discussion

Our findings reveal a picture of unequal disruption — both positive and negative — to PSS brought about by a transition to remote-working, which complements the variability of a wider workforce response (cf. Hobbs, 2021). Working relationships for the most part seem to have remained the same, though there is evidence of remote-working both improving and deteriorating PSS working relationships with academics — albeit the latter is more conspicuous. The extent of their boundary crossing as facilitated by remote-working appears thus only partial.

While a clear majority of respondents declared that their work has become more demanding, just under half identified productivity gains from remote-working. Reported impacts on respondents’ mental and physical health are mainly neutral, and while respondents declare negative impacts, over 20% of our sample reported positive impacts associated with remote-working. While some surveys highlight the deleterious effects of remote-working on mental health (Nuffield 2020, Watermeyer et al., 2021a), our respondents suggest by comparison that campus-based working is no panacea; a view confirmed by pre-pandemic analysis of mental health amongst UK HE staff, which found that PSS accounted for the highest number of referrals to occupational health services (Morrish & Priaulx, 2020). For PSS, as many academics, the pandemic appears to have focused minds on the physical, mental and financial toll of commuting to work and the subsequent relief brought from being able to work from home, while also recognising the value of more flexible working conditions to (attracting and retaining) a diverse work demographic and universities fulfilling an environmental commitment:

Being able to work from home offers more flexibility especially for people with young children, this may enable more new parents to remain in full time employment rather than dropping to part time hours and therefore support career progression for new mothers in particular. Identifying that all services can be provided when working from home has also highlighted the lack of need for office space & transport, therefore hopefully supporting the universities to become more sustainable. (PSS23: Student Engagement, Female, Pre-1992)

Yet, concurrently the challenges of managing remote-working with caring responsibilities while addressed is not nearly so prominent within our respondents’ qualitative accounts — though we note a minority of our sample declare caring responsibilities — particularly when compared with the testimony of UK academics, who have spoken much more extensively of the collateral damage of working from home with child dependents (Staniscuaksi et al., 2020). In fact, our survey evidences little discussion of how, nor immediate indication that spatio-relational changes have been unequally experienced by PSS. Controversially, we might consider PSS as particularly resilient in managing a home and work nexus — a resilience built from they’re being accustomed to inflexible and rigid working practices. Working away from what may be frequently large open-plan office environments is also identified as beneficial to productivity and providing insulation from office politics. Curiously, while a premium tends to be placed on in-person work-based interactions, remote-working is championed by many of our respondents as an enabler of more positive work relationships. We find thus, similarity with other studies of remote-working which evidence its effect in both relieving and escalating stress (De Menezes & Kelliher, 2016).

Our respondents also appear to have good faith in their senior leadership, a finding directly at odds with survey findings of UK academic staff (Watermeyer et al., 2021b). Unlike academics whose professional identity is linked to discipline and scholarly community less than institutional affiliation, PSS arguably possess a stronger institutional attachment as a result of their jobs being more static, less mobile and more directed by management. They are, by comparison, a professional cadre of third space workers, close in character to what Tierney (2006) describes as members of a ‘congenial’ university that exhibit high managerial deference and limited contribution to governance. We might assume then that PSS, as occupiers of the third space, who pre-pandemic were already outside looking in, would find that their limited influence on institutional governance in a remote-working milieu to have further slipped. Conversely it might be the case that their *virtual* connectedness — though in ways discussed, socially inferior — would actually enhance their social capital and powers of advocacy. What we see from the qualitative accounts of our survey respondents is that a transition to remote-working for PSS has benefitted their social capital and availed new spaces of convergence and opportunities for influence, building the basis for stronger trust relationships in universities and beyond. Trust relationships have in such instance cultivated not only by PSS becoming more institutionally prominent via virtual connectedness, but by PSS exploiting remote-working as an opportunity for greater professional autonomy (Watermeyer et al., 2021a). In fact, the potential of a return to campus working is rationalised by respondents as antagonistic to the trust gains provided by the pandemic. However, such a positive disposition to remote-working is not shared by all, and there are those amongst our respondents who are dismissive of trust gains and who view the work-platformisation of PSS as an extension of servitude to their academic counterparts. The potential of trusting relationships in the university is consequently found caught in the balance.

A transition to remote-working, however partial, demands a new paradigm of workplace engagement and interaction, particularly involving university leadership or ‘e-leadership’ (Roman et al., 2019) and rank-and-file staff. This may be challenging yet also advantageous for PSS, who, through an embrace of virtual connectedness, can transcend penalisation by status and social capital, common to the campus-based experience. Our survey of PSS points to the potential of virtual connectedness in generating trusting relationships within universities, and by extension a better integrated, harmonised and we would add, *happy* institutional community. Moreover, we find identified benefits of remote-working to establishing and sustaining professional networks that exceed the isolated campus and complement ideas of virtual work environments enabling global connections and interactions (Cortellazzo et al., 2019).

It may be that much of the success of virtual connectedness through the pandemic is attributable to ‘relatedness’ and the extent to which co-workers have become better known to each other beyond office confines; and have discovered points of commonality and adopted pro-social behaviours in the face of extraordinarily adverse conditions (cf. Okabe-Miyamoto & Lyuobomirsky, 2021). Our survey findings, like other recent studies (cf. Luchetti et al., 2020; Okabe-Miyamoto et al., 2021; Tull et al., 2020), consequently challenge assumptions of the harm caused by physical distancing to social connection, and even demonstrate that a transition to remote-working can be remedial to the isolation and loneliness experienced by some PSS in campus-based settings.

However, our analysis can go further. While we have provided first sight of the experiences of PSS in UK universities during the pandemic, more can be achieved by identifying and analysing commonalities and divergences of these experiences as distinguished by role type and institutional setting. Furthermore, we recommend an investment in longitudinal, internationally comparative and more explicitly qualitative research to provide an ongoing deep dive into the lived experiences of PSS as universities’ continue to (re)calibrate their working practices in a period of what will likely be prolonged post-COVID recovery and incremental transformation.

## Conclusion

While the long-term future organisation of university PSS is unclear, we can at least recognise the emergence of a new narrative of work in universities given impetus by the pandemic as a boundary object, and which talks to integrated and agile forms of working with the potential to transcend the ‘boundary-blocks’ (Watermeyer & Rowe, 2021) of status-based traditions. In what we have presented as the emotional void of virtual connectedness, status and superiority are as valueless as digital etiquette is vague. Work-based polarisation may, therefore, potentially decline, where spaces of ritualistic and performative interactions recede and give way to new spatio-relational dynamics arranged on trust which help to dismantle role prejudices and ameliorate role recognition (Gray, 2015).

However, despite the disruptions of a transition to remote-working caused by the pandemic, the university as a field of interaction may in the longer-term prove resistant to any substantive reorganisation of its professional services. The boundary crossing potential of remote-working may remain untapped in the immediate and post-pandemic context — whenever the latter arrives. An opportunity for a new kind of habitus may thus pass. While there is willingness for a new model of work, the pandemic appears not to have quite delivered the threshold moment to see it through. A spatio-relational value change is shown to be non-conclusive and though many gains from remote-working are apparent, they may not prove sufficiently compelling in the long-run to defeat the innate conservatism of universities and the subjection of PSS to academic patriarchy.
